# Perioperative Outcomes of Surgical Left Atrial Appendage Occlusion During Cardiac Surgery

**DOI:** 10.21470/1678-9741-2025-0299

**Published:** 2026-06-12

**Authors:** Reina Tonegawa-Kuji, Koshiro Kanaoka, Sho Takemoto, Yoko Sumita, Yoshihiro Miyamoto

**Affiliations:** 1 Department of Medical and Health Information Management, National Cerebral and Cardiovascular Center, Suita, Osaka, Japan; 2 Department of Cardiovascular Surgery, Kyusyu University Graduate School of Medical Sciences, Fukuoka, Japan; 3 Open Innovation Center, National Cerebral and Cardiovascular Center, Suita, Osaka, Japan

**Keywords:** Atrial Fibrillation, Thoracic Surgery, Blood Coagulation, Thrombosis.

## Abstract

Surgical left atrial appendage occlusion (S-LAAO) is increasingly performed during cardiac surgery, but perioperative outcomes remain uncertain. Using a nationwide Japanese inpatient database, we analyzed 25,059 adults undergoing valve or coronary bypass surgery (2020 - 2022). After propensity score matching (n = 2,543 each), addition of S-LAAO was not associated with differences in in-hospital mortality, transfusion, reoperation, or 30-day readmission compared to non-S-LAAO group. However, prolonged inotropic support (≥ 2 days) was more frequent with S-LAAO (40.8% vs. 34.7%; odds ratio 1.29, 95% confidence interval: 1.13 - 1.47). Addition of S-LAAO did not increase mortality but was linked to greater inotrope use, warranting further investigation.

## INTRODUCTION

**Table:** 

Abbreviations, Acronyms & Symbols	
AF	= Atrial fibrillation
CI	= Confidence interval
NA	= Not applicable
OR	= Odds ratio
PS	= Propensity score
S-LAAO	= Surgical left atrial appendage occlusion
SD	= Standardized difference

Atrial fibrillation (AF) is a major risk factor for thromboembolic stroke. Concomitant surgical left atrial appendage occlusion (S-LAAO) at the time of cardiac surgery has gained increasing attention as a preventative strategy. A large randomized controlled trial (the Left Atrial Appendage Occlusion Study or LAAOS III) supported the benefit of S-LAAO in patients with AF who underwent coronary artery bypass graft surgery or valve surgeries^[1]^, and the most recent North American guidelines state that S-LAAO is Class I recommended in patients with AF^[2]^. In Japan, an additional reimbursement code for concomitant S-LAAO (K594-4) was established in 2020 for patients undergoing planned cardiac surgery who are preoperatively diagnosed with AF or atrial flutter; however, no reports have described the real-world implementation or perioperative outcomes. The efficacy of prophylactic S-LAAO in patients without AF has also been reported^[3]^. As this practice may become more common, it is increasingly important to investigate strategies to ensure perioperative safety. Therefore, we investigated the effect of S-LAAO during cardiac surgery on perioperative complications, using a nationwide claims database in Japan.

## METHODS

This retrospective cross-sectional study used data from the Japanese Registry of All Cardiac and Vascular Diseases-Diagnosis Procedure Combination (or JROAD-DPC), a nationwide inpatient claims database covering more than 800 Japanese Circulation Society-certified training hospitals during the study period^[4]^. Patients ≥ 18 years old who underwent valve surgery or coronary artery bypass grafting via median sternotomy or minimally invasive cardiac surgery approach between April 2020 and March 2022 were included. We excluded patients admitted in March, the last month of the fiscal year in Japan, to analyze 30-day re-admission. The study was approved by the Institutional Review Board of the National Cerebral and Cardiovascular Center (R23004, April 9^th^, 2023). Categorical data are shown as frequencies (%), and continuous data are presented as medians (interquartile ranges). We performed a propensity score (PS) matching analysis separately to compare in-hospital outcomes between S-LAAO and non-S-LAAO. Multivariate logistic regression models were used to calculate the PS representing the probability of undergoing S-LAAO. Baseline characteristics (Table 1) were used as independent variables. Matching was performed using the nearest neighbor algorithm (1:1 without replacement), with a caliper of width 0.2 standard deviations of the logit of the estimated PS. The absolute value of the standardized difference (10%) was considered a relatively small imbalance. To estimate the odds ratio (OR) and 95% confidence intervals (CIs) for in-hospital complications, mixed-effects logistic regression analysis using the institute as a random intercept was performed. All statistical comparisons were two-sided, and *P* < 0.05 was considered significant. All analyses were performed using STATA 18.0 (StataCorp, College Station, Texas, United States of America).

**Table 1:** Baseline characteristics and results of the study.

	Original cohort			Propensity-scores matched cohort					
	S-LAAO	Non-S-LAAO	*P*-value	S-LAAO	Non-S-LAAO	***P***-value	|SD|	Results of logistic regression analysis	
	N = 2543	N = 22520		N = 2543	N = 2543			Odds ratio (95% CI)	*P*-value
Baseline characteristics									
Age	75 (69 - 79)	72 (65 - 78)	< 0.001	75 (69 - 79)	74 (69 - 79)	0.58	0.02	-	-
Female	1101 (43.3)	9277 (41.2)	0.04	1101 (43.3)	1089 (42.8)	0.73	0.01	-	-
Body mass index	22.3 (19.9 - 24.9)	22.6 (20.2 - 25.1)	< 0.001	22.3 (19.9 - 24.9)	22.2 (20.0 - 24.7)	0.3	0.02	-	-
Emergency hospitalization	4573 (20.3)	433 (17.0)	< 0.001	433 (17.0)	435 (17.1)	0.94	0.00	-	-
Hypertension	2539 (99.8)	22487 (99.9)	0.89	2539 (99.8)	2539 (99.8)	> 0.99	0.00	-	-
Diabetes mellitus	2065 (81.2)	18277 (81.2)	0.96	2065 (81.2)	2085 (82.0)	0.47	0.02	-	-
Dyslipidemia	1125 (44.2)	12081 (53.7)	< 0.001	1125 (44.2)	1130 (44.4)	0.89	0.00	-	-
Chronic pulmonary obstructive disease	111 (4.4)	970 (4.3)	0.93	796 (31.3)	784 (30.8)	0.74	0.01	-	-
Chronic kidney disease without dialysis	177 (7.0)	1257 (5.6)	0.01	177 (7.0)	188 (7.4)	0.55	0.02	-	-
Dialysis	201 (7.9)	2653 (11.8)	< 0.001	201 (7.9)	210 (8.3)	0.64	0.01	-	-
Sinus dysfunction	22 (0.9)	161 (0.7)	0.40	22 (0.9)	19 (0.8)	0.64	0.01	-	-
Heart failure	818 (32.2)	8645 (38.4)	< 0.001	818 (32.2)	808 (32.8)	0.76	0.01	-	-
Ischemic heart disease	406 (16.0)	8490 (37.7)	< 0.001	406 (16.0)	412 (16.2)	0.82	0.01	-	-
Mitral regurgitation	1626 (64.0)	9869 (43.8)	< 0.001	1626 (64.0)	1648 (64.8)	0.52	0.02	-	-
Mitral stenosis	246 (9.7)	878 (3.9)	< 0.001	246 (9.7)	262 (10.3)	0.45	0.02	-	-
Aortic stenosis	593 (23.3)	8207 (36.4)	< 0.001	593 (23.3)	560 (22.0)	0.27	0.03	-	-
Aortic regurgitation	504 (19.8)	5558 (24.7)	< 0.001	504 (19.8)	471 (18.5)	0.24	0.03	-	-
Tricuspid regurgitation	555 (21.8)	2920 (13.0)	< 0.001	555 (21.8)	565 (22.2)	0.74	0.01	-	-
Tricuspid stenosis	2 (0.1)	23 (0.1)	0.72	2 (0.1)	2 (0.1)	> 0.99	0.00	-	-
Pulmonary regurgitation	3 (0.1)	272 (1.2)	< 0.001	3 (0.1)	9 (0.4)	0.08	0.05	-	-
Pulmonary stenosis	0 (0.0)	36 (0.2)	0.04	0 (0.0)	0 (0.0)	NA	NA	-	-
Infective endocarditis	108 (4.3)	1698 (7.5)	< 0.001	108 (4.3)	114 (4.5)	0.68	0.01	-	-
Coronary artery bypass grafting	28 (1.1)	4292 (19.1)	< 0.001	28 (1.1)	29 (1.1)	0.89	0.00	-	-
Valve plasty surgery	762 (30.0)	5698 (25.3)	< 0.001	762 (30.0)	818 (32.2)	0.08	0.05	-	-
Intervention on two or more valves	1551 (61.0)	6749 (30.0)	< 0.001	1551 (61.0)	1552 (61.0)	0.98	0.00	-	-
Outcome									
In-hospital death	88 (3.5)	860 (3.8)	0.37	88 (3.5)	80 (3.2)	0.53	-	1.11 (0.81 - 1.52)	0.51
Transfusion of blood products after the surgery	1116 (43.9)	9533 (42.3)	0.13	1116 (43.9)	1103 (43.4)	0.71	-	1.02 (0.90 - 1.16)	0.74
Mechanical support after the surgery	140 (5.5)	1628 (7.2)	0.001	140 (5.5)	170 (6.7)	0.08	-	0.79 (0.62 - 1.01)	0.06
Reoperation on the next day of the index surgery	10 (0.4)	87 (0.4)	0.96	10 (0.4)	8 (0.3)	0.64	-	1.26 (0.49 - 3.24)	0.63
Catecholamine use for two or more days after the surgery	1037 (40.8)	7323 (32.5)	< 0.001	1037(40.8)	882 (34.7)	< 0.001	-	1.29 (1.13 - 1.48)	< 0.001
Readmission within 30 days of the discharge	125 (4.9)	1168 (5.2)	0.56	125 (4.9)	135 (5.3)	0.52	-	0.92 (0.72 - 1.18)	0.52

CI=confidence interval; NA=not applicable; S-LAAO=surgical left atrial appendage occlusion; SD=standardized difference

## RESULTS AND DISCUSSION

A total of 25,059 patients (41% female) were included; S-LAAO was performed in 2,543 (10.1%). Although the specific valve involved could not be determined, diagnostic codes for mitral regurgitation (64.0% *vs.* 43.8%, *P* < 0.001) and stenosis (9.7% *vs.* 3.9%, *P* < 0.001) were significantly more common with S-LAAO than without it, respectively (Table 1). This is consistent with the clinical practice of performing S-LAAO during mitral valve surgery, typically requiring left atrium opening. Patients who underwent S-LAAO were older than those who did not (75 [69 - 79] *vs.* 72 [65 - 78] years, respectively; *P* < 0.001), and interventions involving more than one valve were more common with S-LAAO (61.0% *vs.* 30.0%, *P* < 0.001). In-hospital death occurred in 3.5% of patients who underwent S-LAAO, comparable to the rate reported in the United States of America^[3]^.

After PS matching, 2,543 patients were included in each group. The standardized differences in the covariates in both cohorts were < 0.1, indicating a well-balanced comparison. Mixed-effects logistic regression analysis in the matched cohort showed no significant association between S-LAAO and in-hospital mortality, transfusion or mechanical circulatory support after surgery (intra-aortic balloon pump, percutaneous axial flow pump, or venoarterial extracorporeal membrane oxygenation), reoperation on the day after the index surgery, and 30-day readmission. S-LAAO was associated with a higher rate of catecholamine use for ≥ 2 days after surgery (40.8% *vs.* 34.7%, OR [95% CI]: 1.29 [1.13 - 1.47], *P* < 0.001). The reason for this remains unclear; however, adding S-LAAO to standard cardiac surgery tends to slightly prolong bypass and cross-clamping times^[1]^. S-LAAO may increase surgical invasiveness and potentially delay postoperative cardiac functional recovery. Further details, such as the dosage and duration of catecholamine use, could not be extracted from the database, highlighting the need for future studies with more granular clinical data.

## CONCLUSION

In summary, in a large nationwide Japanese database, adding S-LAAO to cardiac surgery was not associated with mortality or 30-day readmission, but was significantly associated with prolonged catecholamine use, suggesting the need for further investigation.

## Data Availability

The authors declare that the data supporting the findings of this study are available from the corresponding author upon reasonable request.

## Artificial Intelligence Usage

The authors declare that no artificial intelligence tool was used in the preparation of this article.
